# Unraveling the genetic cause of a consanguineous family with unilateral coloboma and retinoschisis: expanding the phenotypic variability of RAX mutations

**DOI:** 10.1038/s41598-017-09276-0

**Published:** 2017-08-22

**Authors:** Xiu-Feng Huang, Zhi-Qin Huang, Dan Lin, Ma-Li Dai, Qing-Feng Wang, Zhen-Ji Chen, Zi-Bing Jin, Yuqin Wang

**Affiliations:** The Eye Hospital, Wenzhou Medical University, Wenzhou, 325027 China

## Abstract

Ocular coloboma is a common eye malformation arising from incomplete closure of the human optic fissure during development. Multiple genetic mutations contribute to the disease process, showing extensive genetic heterogeneity and complexity of coloboma spectrum diseases. In this study, we aimed to unravel the genetic cause of a consanguineous family with unilateral coloboma and retinoschisis. The subjects were recruited and underwent specialized ophthalmologic clinical examination. A combination of whole exome sequencing (WES), homozygosity mapping, and comprehensive variant analyses was performed to uncover the causative mutation. Only one homozygous mutation (c.113 T > C, p.I38T) in *RAX* gene survived our strict variant filtering process, consistent with an autosomal recessive inheritance pattern. This mutation segregated perfectly in the family and is located in a highly conserved functional domain. Crystal structure modeling indicated that I38T affected the protein structure. We describe a patient from a consanguineous Chinese family with unusual coloboma, proven to harbor a novel *RAX* mutation (c.113 T > C, p.I38T, homozygous), expanding the phenotypic variability of ocular coloboma and *RAX* mutations.

## Introduction

Eye development is a highly intricate and multistep process demanding well-organized genetic events during embryogenesis. Anomalies in eye morphology exhibit diverse severity and can occur in the form of a syndrome or in isolation. Of all eye malformations, ocular coloboma (OC) is a common developmental structural defect defined as a segmental ocular deficiency in the iris, chorioretinal, or optic disc tissue with a prevalence of 2 to 14 per 100000 births^1^, which is usually bilateral and symmetrical. OC can result from the abnormal persistence of the optic fissure in postembryonic life, generally, between the 7 mm and 14 mm stages of embryonic development^2^. In close association with microphthalmia (small eye) and anophthalmia (absent eye)^3, 4^, OC displays a wide spectrum of eye malformations that attribute to approximately 10–15% pediatric blindness^5^.

The genetic etiology of OC related disease is yet to be clearly understood. To the best of our knowledge, mutations, either monogenic or chromosomal, are only responsible for 25–30% of cases^1^. Therefore, genetic causes of a large proportion of ocular coloboma are still unresolved. It is the complexity of eye development that accounts for the variety of structural eye defects, with overwhelming possibilities for disruption. According to a growing body of evidence from genetic studies and animal models, transcription factors and signaling pathways are proven to take a dominant position in fissure closure and optic-cup morphogenesis^3^. A subset of familial or isolated OC cases resulted from mutations in particular genes, commonly in an autosomal recessive or dominant heritance pattern^6–8^. Thus, an unbiased genetic screen and a precise candidate gene approach were indispensable in the molecular diagnosis of OC disease.

In this study, we came across a consanguineous Chinese family in which the proband was clinically diagnosed with unusual ocular coloboma. We combined whole exome sequencing (WES), homozygosity mapping, and thorough variant analysis to dissect the genetic basis, aiming to expand the genotype-phenotype correlations of ocular coloboma.

## Materials and Methods

### Study subjects

The experimental protocol was approved by the Ethics Committee of The Eye Hospital of Wenzhou Medical University and in accordance with the tenets of the Declaration of Helsinki. All experiments were performed in accordance with relevant guidelines and regulations. Informed consent was obtained from all participants. We collected samples from a consanguineous Chinese family from the Eye Hospital of Wenzhou Medical University. Each participant was reviewed by an experienced ophthalmologist and a comprehensive ophthalmic was performed.

We enrolled a consanguineous family referred from the Eye Hospital of Wenzhou Medical University for whole exome sequencing. Blood samples from the proband and his family members were collected and genomic DNA for them was extracted from peripheral blood using a DNA Extraction kit (TIANGEN, Beijing, China) according to the manufacturer’s protocols. A minimum of 3 μg DNA was used in order to construct the exon-enriched DNA library following the manufacturer’s instructions.

### Whole exome sequencing and analyses

Whole exome sequencing was conducted for the DNA sample of the proband patient (IV: 2). The genomic DNA was sheared into 100–300 base pairs and used to generate Illumina libraries in agreement with the manufacturer’s sample preparation protocol, including end-pair, adenylation, and adapter ligation. DNA fragments were captured by hybridization to the capture panel via using the Exome Enrichment V5 Kit (Agilent Technologies, USA) and, sequenced on Illumina HiSeq. 2000 Analyzers for 90 cycles per read following purification and amplification. Briefly, DNA was sheared and subjected to Illumina paired-end DNA library preparation and sequenced using an Illumina HiSeq. 2000 sequencer. After quality control test, the reads were mapped to the reference human genome using SOAPaligner software and SNV and Indel calls were performed using SOAPsnp software and the GATK.

### Homozygosity mapping

Genomic DNA samples were subjected to SNP array analysis using the HumanCoreExome-24 BeadChip (Illumina Inc., USA). Genotyping was carried out according to manufacturer’s instruction including the following steps: DNA digestion, ligation, PCR amplification, fragmentation, labeling, and hybridization. Scanning of the array slide was performed by iScan Reader (Illumina Inc., USA). We performed genome-wide homozygosity mapping using HomozygosityMapper (http://www.homozygositymapper.org/) based on the SNP genotype files.

### Variant analyses and identification

We searched the following database for the presence of the putative disease-causing variants as an initial filtration^9^: Exome Aggregation Consortium (ExAC, http://exac.broadinstitute.org/), NHLBI Exome Sequencing Project (ESP, http://evs.gs.washington.edu/EVS/), 1000 Genome (www.1000genomes.org) and dbSNP137 (www.ncbi.nlm.nih.gov/SNP/). Direct Sanger sequencing was then used to confirm the segregated variants in the present family, using an ABI 3500 Genetic Analyzer (Applied Biosystems, Carlsbad, CA). The effects of the candidate variants were assessed using in silico prediction programs. Missense variants were analyzed by SIFT (http://sift.jcvi.org/), Polyphen-2 (http://genetics.bwh.harvard.edu/pph2/) and MutationTaster (http://mutationtaster.org/). We predicted the topological model for each of the candidate variant polypeptides using web resources including SMART (http://smart.embl.de/) to explore protein domain architectures, and RaptorX structure prediction web-server (http://raptorx.uchicago.edu/) to generate both the wild-type and mutant protein models, visualized any changes in protein folding and structure using PyMol software (vision 1.5). Additionally, multiple sequences were aligned using Clustal Omega (http://www.ebi.ac.uk/Tools/msa/clustalo/) and the grade of conservation of a given nucleotide was evaluated using PhyloP from the UCSC Genome Browser (http://genome.ucsc.edu/).

## Results

### Phenotypic determination

**Figure 1:**
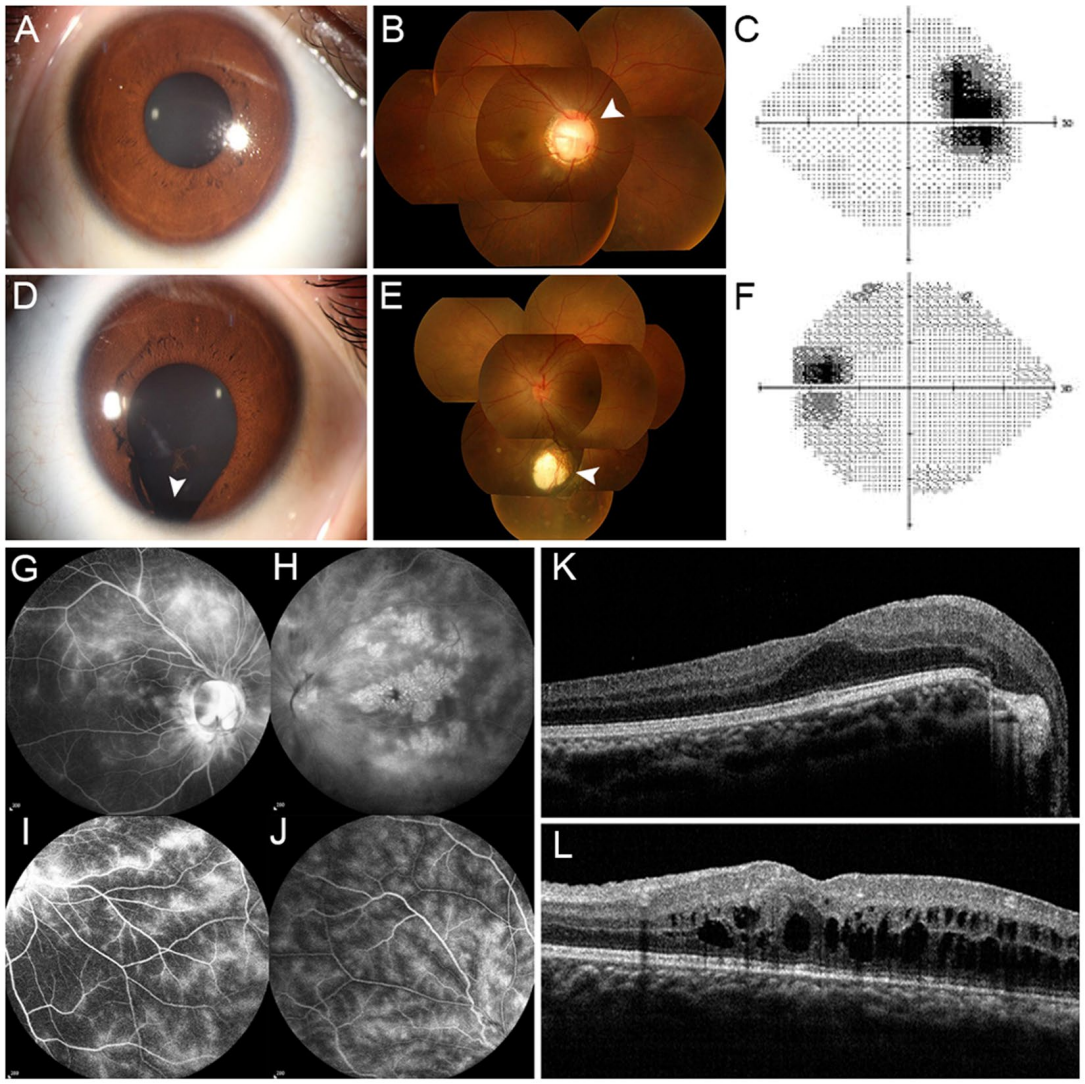
Clinical features of the patient with *RAX* mutation. (**A** and **D**) Representative photographs of the anterior segments. (**A**) The normally-performing right eye. (**D**) The left eye presented with iris coloboma located inferiorly (as indicated by the white arrow). (**B** and **E**) Fundus photographs showed optic disc coloboma in right fundus (white arrow) and choroid coloboma in the left fundus (white arrow). (**C** and **F**) Visual field test demonstrates a physiological blind spot and low visual contrast in the right eye. (**G**–**J**) FFA images revealed leakage of fluorescein from the macular and peripheral retinal telangiectatic vessels in both eyes with different degrees. (**K** and **L**) Spectral-domain optical coherence tomographic and visual field test. (**K**) Macular edema in the right eye. (**L**) Macular edema and retinoschisis in the left eye.

As initial part of ocular coloboma component, a comprehensive ophthalmic examination was conducted in the only affected individual (IV: 2) who was a 14-year-old boy born to the consanguineous family, segregating as an autosomal recessive disorder. Recently he noted a progressive loss of visual acuity in his left eye without any incentive. The examination at his first visit revealed that his visual acuity (VA) was 20/80 OD and 20/200 OS and his best corrected visual acuity (BCVA) was 20/30 OD and 20/200 OS. A clouding of the lens was observed in both eyes of the affected individual, demonstrating minimal cataracts. Slit lamp examination of the anterior segments showed his normal right eye (Fig. 1A), but iris coloboma in the 6 o’clock position was present in left eye (Fig. 1D). Fundus examination revealed an optic disk coloboma in his right eye (Fig. 1B) and choroid coloboma in the left eye (Fig. 1E). Visual fields showed an enlarged physiological blind spot in his right eye (Fig. 1C) and low visual contrast sensitivity in both eyes (Fig. 1C and F). In contrast, no other family members had the diagnosis of OC or complaints indicating eye malformation.

In addition to OC phenotype, results of fundus fluorescein angiography (FFA) and optical coherence tomographic (OCT) suggested bilateral retinal vasculitis and secondary retinoschisis (Fig. 1G, H, K and L). After hormone therapy for 2 months, macular edema in the both eyes gradually abated (Fig. 1I and J).

### WES and homozygosity mapping analysis revealed mutation in RAX

**Figure 2:**
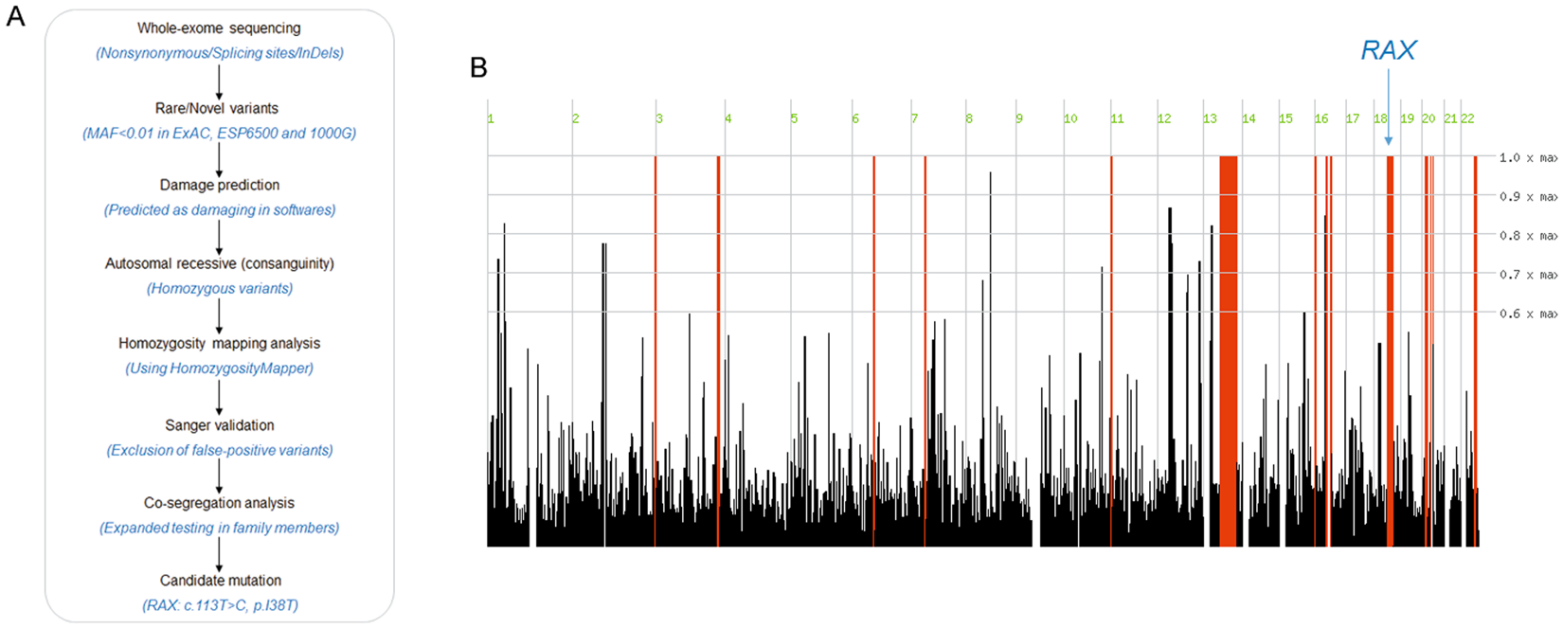
The workflow diagram and homozygosity mapping analyses. (**A**) The workflow diagram. (**B**) The results of homozygosity mapping analyses.

**Figure 3:**
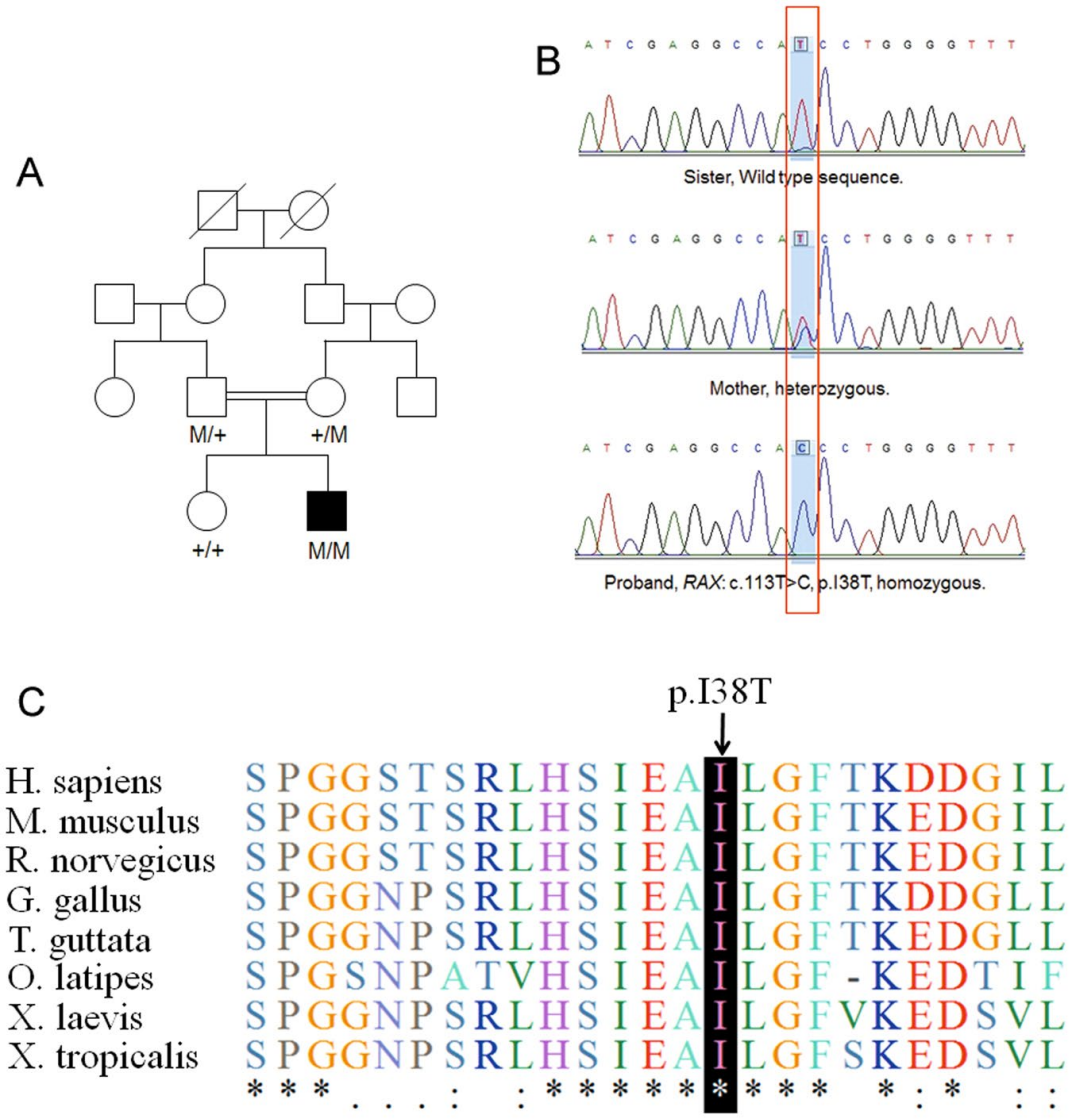
Identification of *RAX* Mutation in the family with ocular coloboma. (**A**) Pedigree. (**B**) Sanger sequencing confirmed the segregation of the homozygous missense mutation, c.113 T > C (p.I38T). (**C**) Evolutionary conservation of amino acid residues Ile38 in RAX. Alignments of the area of novel mutation, in various species, which showed the Isoleucine acid at position 38 is highly conserved. Sequencing alignments performed with Clustal Omega.

Whole exome sequencing was performed in the proband (IV: 2). The mean read depth for the WES was ~30X and the median coverage of the targeted regions reached >95%. The detailed workflow of the variant analyses is displayed in Fig. 2A. Variants with minor allele frequency (MAF) of >0.005 in any of the variant databases were excluded. Due to consanguinity between the parents, an autosomal recessive inheritance pattern was considered first. Homozygosity mapping analysis was performed based on the proband’s SNP genotype files using HomozygosityMapper. The results showed that a total of 12 loci of homozygosity were larger than 2 Mb (Fig. 2B). After employing a step-by-step filtering strategy (Fig. 2A), there are three candidate variants left in homozygous regions (*RAX*, *TRPM5* and *PCDH17*). Only one missense mutation in *RAX* (c.113 T > C, p.I38T) survived after expanded testing in family members (Fig. 3A–B).

The missense mutation I38T was absent in dbSNP137, 1000 G, ESP6500 and an in-house database consisting of 500 Chinese exomes, as well as in the ExAC database. Additionally, homozygosity mapping analysis demonstrated that *RAX* gene is located in a large homozygous region on chromosome 18 that encompasses 18.19 Mb (Fig. 2B). The variant c.113 T > C resulted in a switch from a hydrophobic amino acid (isoleucine) to a hydrophilic amino acid (threonine). The genotype segregated with the phenotype in this family (Fig. 3A, B). Parents are heterozygous carrier while the healthy sister does not harbor this mutation. The isoleucine at position 38 is highly conserved up to tropicalis (Fig. 3C) and the nucleotide position c.113 T > C has a PhyloP score of 2.986. Taken together, we identified a homozygous mutation c.113 T > C (p.I38T) in *RAX* gene responsible for ocular coloboma.

### Comparative and structural analyses of RAX mutation

**Figure 4:**
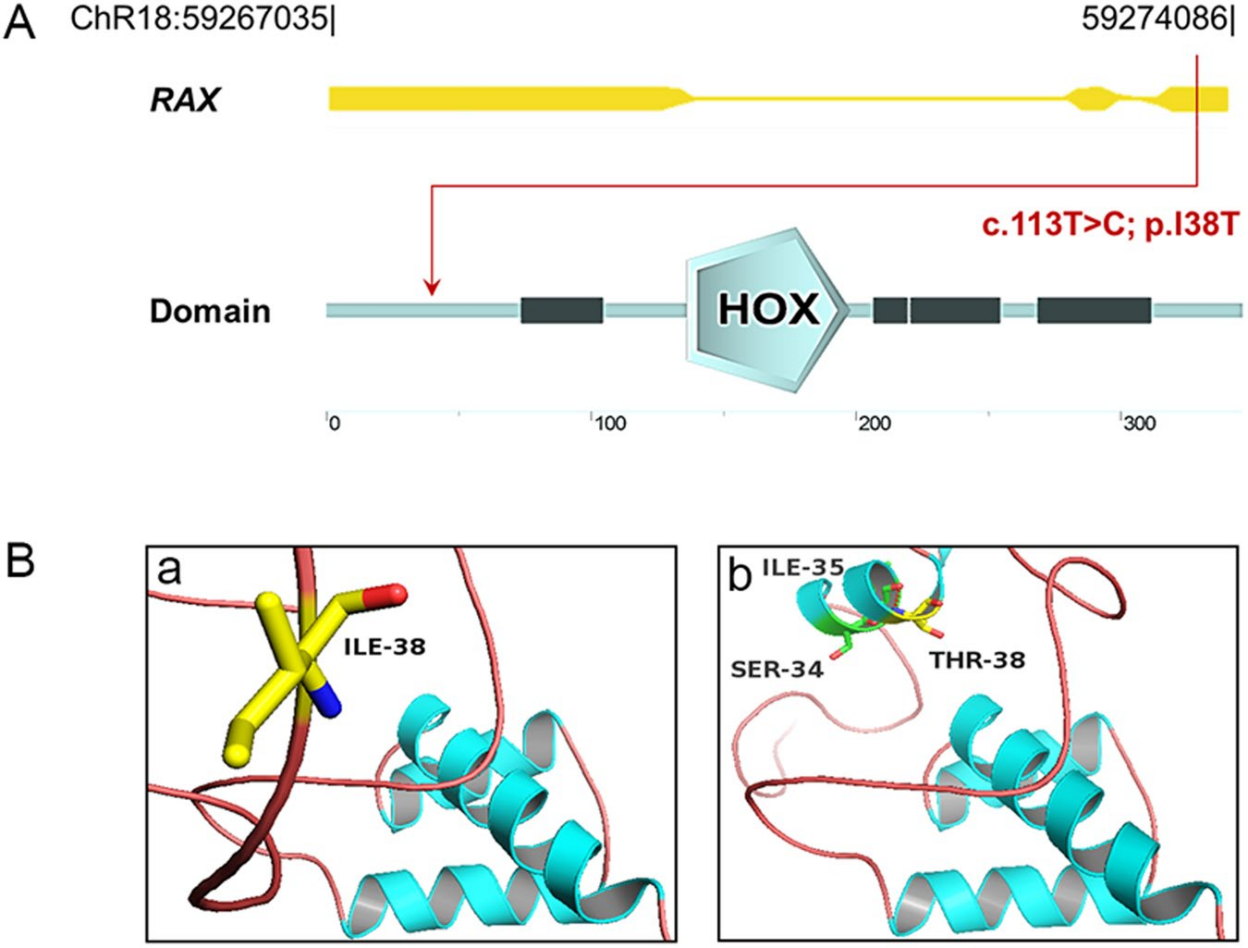
Modeling structure analyses of *RAX* mutation. (**A**) Map of 7.05 kb RAX gene. Predicted RAX domain structure. Homeobox is indicated in light blue and low complexity region is indicated in dark blue. (**B**) Predicted crystal structure of the RAX wide type and mutant type including homeobox domain of RAX (residue136-198). A view of residue 38 highlighting the WT (isoleucine) and mutated (threonine) at the position is in the two bottom boxes.

The proband was found to carry a mutation within exon 1 on both alleles of the *RAX* gene (Fig. 4A). The c.113 T > C mutation results in a missense amino acid change, isoleucine to threonine, at residue 38 (I38T) of RAX. Additionally, the amino acid I38 is located in an Octapeptide motif (http://www.uniprot.org/uniprot/Q9Y2V3). In silico analysis including SIFT and MutationTaster predicted the missense mutation I38T to be damaging (SIFT score: 0; MutationTaster score: 1) and PolyPhen2 predicted it to be possibly damaging (PolyPhen2 score: 0.873) to the protein, retina and anterior neural fold homeobox (Protein Accession: Q9Y2V3). Subdomain identification was performed using SMART, indicating this mutation neither located in homeobox nor paired box. The potential effect on RAX function was further analyzed using the RaptorX service, visualized using PyMol (Fig. 4B). Structural modeling was built based on the crystal structure of the template 2m0cA (p-value 2.51e^−04^), in which total 138 residues were modeled in the RAX structure. The sequence alteration causes a gain of helical conformation for the residue I38, which we postulate could induce protein spatial structure alteration of RAX. Together with the genetic analysis, the structural analysis supports the putative causality of *RAX* I38T.

## Discussion

In the present study, we successfully carried out molecular genetic testing on a consanguineous family with unilateral coloboma and retinoschisis. Using the combination of whole exome sequencing and homozygosity mapping, we identified a novel mutation (c.113 T > C, p.I38T) in *RAX* gene with autosomal recessive transmission. This combined approach has proven to be highly efficient in the identification of novel recessive genes of ocular disease^10–14^. Based on the consanguineous pedigree and efficient methodology, we uncovered the disease-causing mutation in *RAX*, which is the only candidate surviving our stringent filtering process. Our results are further supported by clinical, functional and modeling data, all of which provide a validation for OC disease pathogenesis.

Genetic components are generally considered to make more significant contributions than potential environmental factors in the etiology of congenital ocular malformations^15, 16^. Several genes have been implicated to establish some association with developmental eye defect, including *PAX6, RAX, SOX2, OTX2, CHX10, PAX2, SHH, SIX6* and *et al*.^1, 7^. Of all these genes, the retina and anterior neural fold homeobox (*RAX)* gene encodes a homeobox-containing transcription factor that occupies a critical role in human and vertebrate eye formation by regulating the early eye specification and subsequent proliferation, supported by multiple lines of evidence from animal model^17, 18^. *RAX* is a paired-like homeobox-containing gene initially expressed in the anterior region of developing embryos, and later in the ventral hypothalamus and retina^19^. Knockdown of *RAX* during early eye development in Xenopus and zebrafish could lead to microphthalmia or anophthalmia while knockdown of it in mature retina could induce photoreceptor degeneration^20^. In contrast, overexpression of *RAX* could give rise to extra or ectopic retinal tissue^21, 22^. Despite animal studies have established *RAX* as a high-order gene in eye development^23, 24^, only few reports of *RAX* mutations in ocular dysgenesis have been reported in human subjects, and these include anophthalmia, microphthalmia, and ocular coloboma^1, 3, 7, 8, 18, 25–27^. For instance, Voronina *et al*. identified one boy out of 75 individuals with microphthalmia/anophthalmia as a compound heterozygote for *RAX* mutations within the DNA-binding homeodomain^27^.

The enormous phenotypic and genotypic heterogeneity make it challenging to perform clinical genetic testing for patients with coloboma. Thus, genetic causes of a large proportion of coloboma are still unresolved. The clinical features and biometric data in the eyes of children with coloboma have been described in previous study. Five cases had co-occurrence of iris and choroid coloboma out of an examination of 196 children^5^. In addition, coloboma usually occurs bilateral and symmetrical. Intriguingly, the case involved in our study displayed iris coloboma in left eye and choroid coloboma in right eye, showing a rarely reported case. Nikolas *et al*. once described a patient with optic nerve coloboma OD only who possessed a heterozygous missense mutation in *RAX*, suggesting the mild nature of the colobomatous defect that *RAX* mutation carriers can harbor^7^. In this study, we report a homozygous missense mutation in exon 1 of *RAX* in a patient with iris coloboma OS and optic disk coloboma OD, consistent with the hypothesis that mutations on both alleles could cause more severe phenotype. The possibilities of copy number variations and uncovered regions were excluded^28, 29^. Our findings provide a further understanding on the role of the *RAX* mutation in OC disease pathogenesis.

In conclusion, combination of WES and homozygosity mapping identified a novel homozygous *RAX* mutation in a consanguineous family segregating with rarely reported asymmetrical coloboma. Clinical findings and genetic results support that *RAX* mutation is responsible for eye malformations. The novel mutation revealed by our group not only proves that WES and homozygosity mapping is a useful tool for dissecting pathogenic mutations in patients with genetically heterogeneous diseases, but also enrich our understanding of the molecular basis of ocular coloboma, as well as genotype-phenotype correlation.
